# Fitness Soccer Athletes Training at the University of Limpopo, South Africa: Are the Macronutrients Intake and Anthropometric Status of These Athletes Optimal?

**DOI:** 10.3390/ijerph191912650

**Published:** 2022-10-03

**Authors:** Masodi Makhafola, Hendrick Makhubela, Sylven Masoga, Sefora Hazel Makuse

**Affiliations:** Department of Human Nutrition and Dietetics, University of Limpopo, Sovenga 0727, South Africa

**Keywords:** energy, macronutrients, body mass index, recommendations, soccer athletes

## Abstract

Background: Dietary practices of the University of Limpopo soccer team athletes have been reported. However, the practices of those engaging in soccer for general fitness from different non-competitive teams remain unknown. To respond to this gap, the researchers investigated the energy, macronutrient intake, and BMI status and further correlated the two variables of the fitness non-competitive soccer athletes registered at the University of Limpopo in South Africa. Method: A quantitative study design was undertaken to conveniently sample 60 out of 90 fitness soccer athletes from the four non-competitive soccer teams at the University of Limpopo sports grounds. Ethics approval was obtained from the University of Limpopo Research and Ethics Committee, and permission was given by the university sports management and team coaches. Athletes signed the informed consent form before participating in the study. Data were collected at the soccer fields during the afternoons before the start of training. Macronutrient intake data were collected using multiple (two) 24-h recall questionnaires on different days, which were validated by the food frequency questionnaire. Weight and height were measured using a digital scale (Seca 813 electronic flat scale) and stadiometer (Seca 213 portable stadiometer) for BMI calculations, respectively. The average energy and macronutrient intakes were calculated through the SAMRC FoodFinder software (3.0). The nutrient averages, together with the BMI results, were loaded into SPSS (26.0) for further analysis. Descriptive statistics were used to report the energy, macronutrient intake, and BMI statuses of athletes using percentages, means, and standard deviations (±SD). A one-way ANOVA test was used to determine the association between the latter variables. A *p*-value of ≤ 0.05 was the criterion used to correlate the variables. Results: All (100%) athletes were males, most of whom played soccer for 2–3 years while at the university. Almost half (48%) of athletes consumed energy (39.6 kcal/kg) below the recommendations. About 92% and 53% of athletes consumed carbohydrates (5.0 g/kg) and fat (1.2 g/kg) below the recommended values; while 43% consumed protein (1.4 g/kg) optimally. The majority (>80%) had a normal BMI (21.6 ± 2.6 kg/m^2^) status. However, there were no relationships between the energy (*p* = 0.383), CHO (*p* = 0.261), protein (*p* = 0.543), and fat (*p* = 0179) intake and the BMI status of athletes. Conclusion: The macronutrient intake of fitness soccer athletes at the University of Limpopo is, on the whole, suboptimal. However, the athletes had normal body weights. There was no association between both the energy and macronutrient intake and the anthropometric (BMI) status of soccer athletes.

## 1. Introduction

Soccer is the most popular sport that is physically and technically demanding [1]. The sport requires proper and optimal dietary intake to support endurance training [2]. The daily energy requirements are increased for this sport due to the type, intensity, and duration of training [3]. Therefore, nutrition becomes vital in supporting athletes during training and competition days; and even for general wellbeing [4]. Optimal carbohydrate (CHO) and protein intakes are important to sustain the duration of sports performance and adequate protein synthesis, respectively [5]. Even though soccer is a global sport [4], little research, especially around macronutrient intake and anthropometric status, has been devoted to this sport. The current research identified a similar gap among fitness student soccer athletes registered at the University of Limpopo (UL). A single study attempted to address this concern, suggesting that the dietary practices of UL soccer athletes were deviating from standards and highlighted, among other things, a need for improved adherence to nutritional recommendations [6]. The latter study, however, focused primarily on the dietary practices of the UL competitive soccer team (squad) and not the fitness (non-competitive) athletes. These non-competitive athletes are registered students of UL and members of several teams participating in soccer, primarily for general fitness or health maintenance purposes. However, the UL competitive soccer team is often constituted from these existing teams of non-competitive members training at the UL sports grounds. Given the profession of the prior researchers (dietetics), it is thought that optimal nutrition and staying healthy during sports participation, regardless of the engagement goal, remains significant. Therefore, building on the findings by Masoga et al. (2021), the current research focused on the non-competitive soccer athletes who are often overlooked during the provision of health-related programmes such as nutrition services. The athletes’ meals somewhat deviate from the general health and soccer sports recommendations as they consist mostly of fast foods such as bunny chow, fried chips, and high quantities of starch (maize meal) with offal. These meals may encourage excessive consumption of some macronutrients, for instance, CHO or fats, at the expense of others, which may predispose athletes to impaired soccer performance and/or undesired anthropometry. Therefore, the significance of the current study was to investigate the energy, macronutrient intake, and BMI status of fitness non-competitive soccer athletes at UL to encourage adherence to good nutrition while involved in sports. This research intends to highlight the significant inclusion of sports nutrition practitioners within the sport of soccer in general and within institutions of learning.

### 1.1. Energy

Energy is generally required for the control and maintenance of bone density, endocrine functioning, and proper immunity [7]. Energy requirements for soccer athletes are higher, given the nature of the sport [8]. Therefore, to avoid deficits, energy amounts of 25–35 kcal/kg/day should be adequate for soccer sports [7]. The International Society of Sports Nutrition (ISSN), however, recommends higher amounts of 40–70 kcal/kg/day [8] for athletes training more than five times per week, three hours per day. Alternatively, the use of the Harris–Benedict equation [Males: BEE = 66.5 + (13.8 × Weight) + (5 × Height) − (6.8 × Age)] and an activity factor of 1.8–2.3 may be applied in determining the energy needs for soccer athletes [9].

### 1.2. Carbohydrates

Carbohydrates (CHO) consumption is important to enhance the performance of soccer athletes during matches [10]. This macronutrient is regarded as key during soccer practices and competitions [4]. The intake of CHO during training should be determined according to the characteristics of the event. Therefore, the American Dietetic Association (ADA) and the Union of European Football Association (UEFA) expert group generally recommend CHO amounts of 6–10 g/kg/day [11] and 6–8 g/kg/day for light fixtures soccer training, respectively [4]. To maximize glycogen storage [12], the ISSN encourages athletes to consume 8–10 g/kg/day of CHO [13]. Three to four hours before the training, athletes are encouraged to consume 200–300 g of CHO [14]. The pre-training meal should be minimal in saturated fat and fiber to avoid delays in gastric emptying, which may induce bloating and nausea. However, the ISSN encourages the intake values to be expressed in g/kg with amounts ranging from 1–2 g/kg of CHO 2–3 h before training [15]. Given the nature of soccer training, which usually lasts up to 2 h or more, athletes should consume glucose amounts of 30–60 g/h (or 6–8% of CHO solution) during the performance [15]. Lastly, an amount equivalent to 1.0–1.5 g/kg of CHO immediately after training in the first 30 min and, thereafter, every 2 h for 4–6 h is recommended [8,14]. These amounts should consist of an adequate combination of fluids, electrolytes, energy, and CHO [15]. On the other hand, the ISSN recommends 1.5 g/kg BW or 0.6–1.0 g/kg of BW during the first 30 min and, again, every 2 h for 4–6 h within 30 min post-training [15]. In general, meals or sports drinks containing CHO and electrolytes should be ingested before, during, and after exercise to assist in maintaining blood glucose concentration, supply fuel for muscles, and decrease the risk of hyponatremia and dehydration [9]. A higher CHO diet (>60% of energy) consumed during the training periods and the week leading to the competition is associated with enhanced muscle glycogen concentrations, thus improving the athlete’s performance [9].

### 1.3. Protein

Similar to CHO, protein needs must be carefully considered during periods of high physical activity for optimal body weight, replenishment of glycogen stores, and delivery of satisfactory protein to build and mend tissue [9]. Therefore, a protein amount of 1.2–2.0 g/kg/day is recommended to support metabolic adaptation and protein turnover [11]. However, the ISSN recommends a slightly higher protein amount of 1.4–2.0 g/kg/day to optimise training-induced adaptations [16]. On the other hand, the UEFA expert group (2020) suggests an intake of up to 2.2 g/kg/day during soccer match training. However, protein intakes above 2.0 g/kg/day are generally associated with ureagenesis, osteoporosis, and renal failure later in life [6]. In instances where protein supplements may be required, 0.3–0.4 g/kg of creatine is recommended [4]. However, whenever athletes can meet daily adequate protein needs through a diet, supplementation may not be necessary. For pre-training sessions, protein amounts of 0.15–0.25 g/kg taken with CHO foods are recommended [8,15], while an intake of 0.2–0.5 g/kg is recommended during the post-training periods [13,15]. The quality of protein with a preferable high biological value group is recommended for the supply of essential amino acids [4].

### 1.4. Fat

Dietary fat is a critical component of the training diet for athletes as it is a source of energy and facilitates the absorption of fat-soluble vitamins [5]. However, the required amount of fat depends primarily on the soccer athlete’s training goals. Extreme fat restriction is discouraged for endurance sports like soccer as this may result in inadequate energy intake, leading to fatigue, poor performance, and a greater risk for illness or injury [17]. Generally, the focus on fat should be more on the type than the amount. For instance, higher consumption of omega-3 polyunsaturated fatty acids and reduced intake of saturated and trans fats is encouraged [17]. On the whole, the fat recommendation in sports is similar to or slightly higher than that of the general population, suggesting 20–35% of TEE [4,8].

Overall, the consumption of macronutrients for soccer athletes should be spread throughout 4–6 meals per day, especially during the day of training. The summary of energy and macronutrient recommendations for soccer athletes during training is presented in Table 1.

### 1.5. Anthropometry (Body Weight)

Body size, composition, and/or body weight contribute toward an athlete’s potential for success in any given sport [9,16], including soccer [18]. Bodyweight can influence an athlete’s speed, endurance, and power, whereas body composition can affect an athlete’s strength, agility, and appearance. A lean body, such as one with a greater muscle/fat ratio, is often advantageous in sports where speed is involved [9]. Given that BMI fails to distinguish between lean and fat mass, caution should be taken when interpreting the results of this tool for athletes [19]. Optimal dietary intake is necessary for the optimal anthropometric status to enhance the performance of soccer athletes [18]. The summary for BMI categories according to the Centers for Disease Control and Prevention (CDC) is given in Table 2.

## 2. Material and Methods

### 2.1. Participants

The participants were registered students at the UL participating in soccer sports for fitness purposes. These non-competitive athletes usually train in the afternoons at the UL sports soccer grounds. The university has two soccer grounds and one stadium (Oscar Mpetha), which these athletes utilise. At the time of data collection, there were 90 soccer athletes belonging to four non-competitive teams training at the university grounds. All the athletes were registered students in various learning programmes at the UL.

### 2.2. Research Methods and Descriptions

A quantitative descriptive study design was adopted to conveniently obtain sixty (60) out of the ninety (90) fitness (non-competitive) soccer athletes from the four soccer teams that were available at the UL sports grounds during the time of data collection. The athletes were approached by the researchers while about to engage in training at the UL sports grounds in the afternoon. During recruitment, a research information document containing details about the study was provided to all fitness soccer athletes two weeks before the commencement of research. Data collection was thereafter conducted, using 60 out of 90 athletes who responded, giving a response rate of 67%. A convenient sampling technique was used as data were collected during a period when COVID-19 lockdown restrictions were eased to allow limited participation in sports activities between the months of October and November 2020. All COVID-19 regulations were adhered to during data collection. Ethical clearance was obtained from the Turfloop Research and Ethics Committee (TREC) (TREC/254/2020:UG) before data collection. Permission to conduct the study was sought from the UL sports management section and soccer coaches. Athletes signed the informed consent forms before participating in the study. Data were collected for 5–10 days at the two soccer sports grounds in the afternoons, three hours before the commencement of the training. Demographic information was collected through a self-completed questionnaire. The following demographics were included (but not limited to): the age and gender of athletes and the duration and frequency of training in a week. Dietary intake, which included the record of the food items consumed in the previous day, was collected using a quantified 24-h recall questionnaire. Marked household measurement utensils such as spoons, cups, and bowls were used to assist athletes in recalling the estimates of food portions consumed in the past. Food mannequins from Nasco Nutrition were used to simulate a plate and for athletes to identify certain food items consumed. Additional techniques used to assist athletes during the recall process included the probing of incidences athletes engaged in during the previous day, for instance, the music and learning activities conducted. The 24-h recall questionnaire was collected on two different days, for example, Thursdays and Mondays, to cover food consumption patterns during the weekdays and over the weekend, respectively. The food frequency questionnaire (FFQ) was then used to determine the consumption frequency of certain food items of food groups over a particular period. The questionnaire was also used to validate portion sizes or quantities of food items appearing on the athletes’ 24-h recall questionnaires. The anthropometric measurements of the athletes were conducted by trained researchers on anthropometry techniques. In measuring the weights of the athletes, the Seca 813 mobile electronic fat scale was used. Athletes were requested to first void their bladders, remove soccer boots to be barefooted, and remain in their lightest training clothes. The scale was placed on a flat surface, calibrated, and zeroed before the weights were measured. Standing height was measured using the Seca 213 portable stadiometer, which was also placed on a flat surface. The athletes stood upright to maintain the Frankfurt plane position with arms flexed to the sides. The measurements of height were taken at full inspiration. All the weight and height measurements were recorded to the nearest 0.1 kg and 0.1 m to calculate the BMI [weight (kg)/height (m^2^)].

### 2.3. Statistical Analysis

Energy and macronutrient intakes from the multiple 24-h recall questionnaires were loaded into the South African Medical Research Council (SAMRC) FoodFinder (version 3) (SAMRC, Cape Town, South Africa) to determine value intakes. The average of the two recall questionnaires was considered for dietary intake. The collected data were captured in excel and then exported to SPSS (version 26.0) (IBM, Armonk, NY, USA) for further analysis. Descriptive statistics such as summary statistics (percentages), measures of central tendency (mean values), and the dispersion of the data (minimum and maximum standard deviations [±SD] values) were used to describe the demography, intake of energy, macronutrient, and BMI status of the athletes. The energy and macronutrient results were then compared to the ISSN (2018) standards to reflect athletes consuming macronutrients below, within, and above recommendations (Table 1). The BMI values of athletes were also compared with the standards stipulated by the CDC [20] to reflect athletes falling below, within, and above given ranges. In determining the relationship between the variables, a one-way ANOVA test was used with a probability value (*p*) of ≤0.05 as the criterion significant to associate energy and macronutrient intake and the anthropometric status of the athletes.

## 3. Results

Demography

All the 60 soccer athletes (100%) in our study were African males, with a mean age of 21.6 (±2.01) years. All athletes were registered students at the UL, the majority (89%) of whom resided within the university residential premises. More than half of the athletes (67%) participated in the soccer sport for 3 years while registered at UL. The majority of these athletes (67%) trained once a day for almost 1½ h at UL sports grounds. The summary of the demographic results is presented in Table 3.

**Figure 1:**
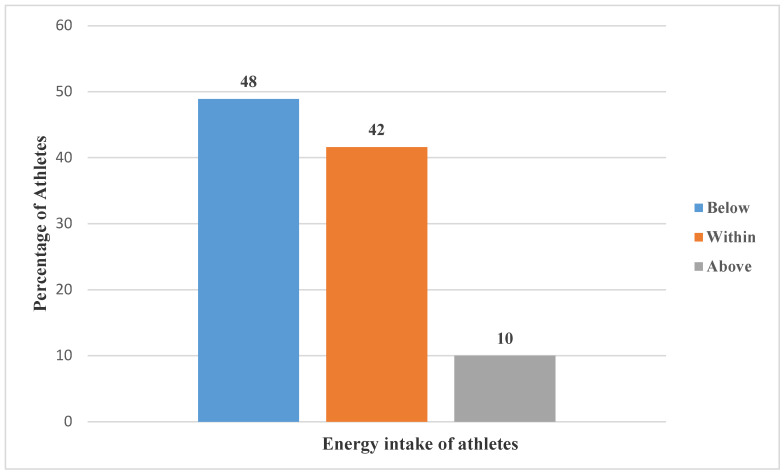
Energy intake of athletes.

**Figure 2:**
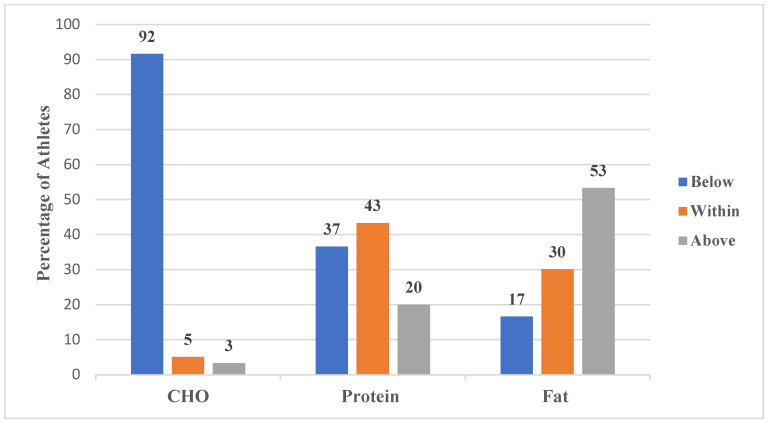
Macronutrient intake of athletes.

**Figure 3:**
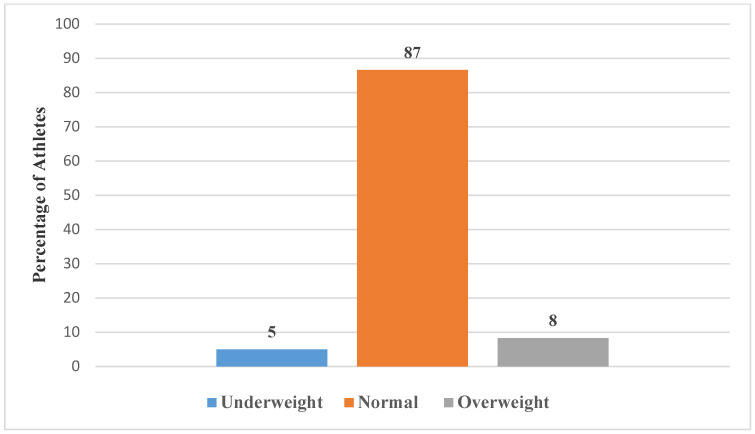
Anthropometry of athletes.

The energy, macronutrients, and BMI status of these soccer athletes are presented in Figure 1, Figure 2 and Figure 3.

The figure above illustrates the energy intake of athletes. Almost half of the group (48%) consumed energy (39.6 ± 12.3 kcal/kg/day) below the recommendations, while 42% and 10% of the athletes consumed energy within and above the recommendations, respectively.

The figure above illustrates the CHO (5.0 ± 1.5 g/kg/day), protein (1.4 ± 0.4 g/kg), and fat (1.2 ± 0.6 g/kg/day) intake of athletes. The majority of the athletes (92% and 53%) consumed CHO and fat below and above the soccer sport recommendations (respectively). The majority of athletes (43%) consumed protein within recommendations. A few athletes (3% and 20%) consumed some macronutrients (CHO and protein) above the recommendations.

Table 4 illustrates the energy and macronutrients intake of the whole group of athletes. On the whole, the intake of energy and CHO was suboptimal while that of protein and fat was within and above recommendations, respectively.

Figure 3 illustrates the BMI status of athletes. The mean weight was 65.0 ± 8.1 kg placing the majority (87%) of the athletes with a normal BMI (21.6 ± 2.6 kg/m^2^).

Table 5 illustrates the relationship between BMI and macronutrients intake per each BMI category. There was no relationship found between the anthropometric status and the dietary intake of soccer athletes.

## 4. Discussion

### 4.1. Energy and Carbohydrates

In our study, the majority of soccer athletes consumed energy (39.6 kcal/kg/day) and CHO (5.0 g/kg/day) below the recommendations. A similar intake of energy below recommended values was reported by Jenner et al. [21] among Australian soccer athletes. Another study in Limpopo province reported the same findings among the athletes, although the focus of this study was on a different group of athletes, bodybuilders [22]. However, in another study by Steffl et al. [23], adequate energy intake was reported among athletes. The athletes in the current study were registered students of the UL, and there is a possibility that most of their income may have been dedicated to tuition. This could be factual as most athletes consumed a few meals during the day. Additionally, the majority of the athletes skipped breakfast and lunch, possibly due to the “tight” academic schedules, for instance, practical exposure. The researchers further postulate that these soccer athletes may have lacked nutrition guidance during their sports as there is no dedicated nutrition practitioner for soccer. In Limpopo Province, starchy foods, for example, maize meal, are generally known to be consumed in excessive quantities by most Africans. Therefore, athletes may have intentionally attempted to impress researchers by under-reporting their portion sizes, given our profession (dietetics). It could also be possible that not all food items were remembered by athletes during the recall process [24]. However, regular suboptimal consumption of energy and/or CHO quantities may negatively impact soccer athletes’ performance outcomes [25].

### 4.2. Protein

Soccer athletes should consume sufficient protein to ensure adequate protein synthesis and training recovery [5]. In our study, almost half of the athletes consumed protein (1.4 g/kg) within the soccer recommendations. These results are comparable to the Australian soccer athletes’ results, where half of the athletes consumed protein within the recommendations [2]. On the contrary, another study involving professional soccer athletes reported high protein intakes above recommendations [26]. The latter was reported in a few of the athletes within our study. The excessive protein intake may be due to the belief that high protein intake enhances muscle bulkiness which could possibly positively impact sports performance [26]. A higher protein intake is thought to prompt several health risks [6]. On the other hand, the low protein intake by soccer athletes in our study may be due to the athletes solely not consuming enough energy intake directly, affecting the protein utilisation for energy supply.

### 4.3. Fat

For athletes to provide the essential fatty acids, facilitate absorption of fat-soluble vitamins, and contribute energy for weight maintenance, adequate fat should be consumed [9]. More than half of the athletes in our study consumed fat (1.2 g/kg/day) above the recommendations. These findings are contrary to those in a study among athletes registered at Charles University (Czech Republic) who consumed fat within the recommendation [23]. However, a study involving the two Scottish Premier League clubs during the competitive season reported similar findings to those of our research. The majority of the soccer athletes in the latter study consumed fat above the recommendations [27]. The results by Maughan are incomparable to our study as athletes in our study are engaging in soccer for fitness and not necessarily competing at a professional level. The results from our study confirmed the observation that the meals of fitness soccer athletes are composed mostly of fatty foods. Although food sources that are known to contain more oils and fats, for instance, margarine and cooking oil, appeared least on the multiple 24-h recall questionnaires for most athletes, we suspect that intakes could have been under-reported with the intention to impress the researchers. Still, the quantity of the required fat depends chiefly on the soccer athlete’s goals and training schedule [5].

### 4.4. Body Mass Index

More than two-thirds of the soccer athletes in our study had normal body weights, 21.6 ± 2.6 kg/m^2^. Our findings are similar to those reported by Popovich et al. [28] in an intervention study of anthropometric measurement and body composition among soccer athletes. The control group of the latter study had a significantly higher BMI when compared with the intervention (24.1 ± 1.1 kg/m^2^). In another study by Gardesevic et al. [29], soccer athletes’ anthropometric status was studied, and normal BMIs were reported. The latter could have been contributed by the intensity of engagement in soccer training, the number of years athletes participated in soccer, and/or adherence to a proper diet. In the current study, a few (5% and 8%) of the athletes were either underweight or overweight with the majority presenting normal weights. These findings potentially are very doubtful as these soccer athletes consumed a few meals during the day, given their academic and practical schedules. In addition to this, some athletes skipped meals, particularly breakfast and lunch. Meal frequency may be used to maintain weight among athletes [13]. The BMI status of our soccer athletes could, however, be supported by the fact that most athletes participated in soccer sports for three years or more, training for almost the whole week. This could have possibly impacted positively the weight status of these athletes. However, in sports, caution is needed when interpreting BMI as a screening tool. This tool may not reflect accurate results as it fails to distinguish the percentage of lean body mass from fat mass [30].

Although the intake of some macronutrients and energy of the athletes was generally suboptimal, our study found no significant correlation between the BMI status and the athletes’ dietary intake (Table 5). We postulate that these athletes had limited or restricted time to consume meals that were adequate to meet their daily energy and some macronutrient requirements, given the commonly known nature of university programmes. However, a study conducted in Cape Town (Stellenbosch University) in South Africa (SA) found a significant correlation between normal BMI and the consumption of adequate macronutrients by athletes [31].

## 5. Recommendations

Athletes may have overlooked the importance of achieving optimal nutrition while involved in soccer. Therefore, we recommend empowering these and other soccer athletes at the UL through the appointment of a nutrition expert for frequent sessional visits and nutritional intervention. This will assist soccer athletes in becoming nutritionally conscious and adhering to sports nutrition guidelines as much as possible. In this way, athletes will be able to identify foods that will directly support sports performance while at the same time maintaining optimal intake. Further studies on the nutrition knowledge and percentage of body fat of soccer athletes are, however, warranted to further identify the support required by this group.

## 6. Strengths and Limitations of the Study

This study is one of a few focusing on the dietary practices and anthropometric profiles of university soccer athletes in Limpopo Province. The dietary collection tools used in this research were validated during the National Food Consumption survey (2005). Data were collected during a period when COVD-19 lockdown restrictions were eased down to different levels, and a few students were just repatriated back to the University of Limpopo. This contributed to smaller sample size (lower response rate of 67%) in this research. Therefore, the findings of this study cannot be generalised to the entire soccer sports athletes within other universities.

## 7. Conclusions

The aim of the research study was to determine the macronutrients intake and anthropometric status of fitness non-competitive soccer athletes at UL, SA. The athletes’ energy and CHO were suboptimal, while the protein and fat were optimal and above the recommendations, respectively. Athletes’ anthropometric status (BMI) was, however, normal. There was no correlation found between the athletes’ energy and macronutrients intake and the anthropometric status (BMI) of these fitness soccer athletes.

## Figures and Tables

**Table 1:** Energy and macronutrient recommendations [8].

Energy	kcal/kg/Day	40–70
CHO 8–10 g/kg/day	Pre-training	1–4 g/kg (3–4 h before training)
	During-training	6–8% Dextrose
	Post-training	1–1.5 g/kg (first 30 min after training) 100 g in the next 2–4 h after training
Protein 1.4–2.0 g/kg/day	Pre-training	0.12–0.25 g/kg
	During-training	No specific recommendation
	Post-training	0.15–0.25 g/kg
Fat 0.5–1 g/kg/day	Pre-training	Avoid excessive intake
	During-training	Avoid intake
	Post-training	No specific recommendation

**Table 2:** Body Mass Index classifications [20].

Interpretation	Classification (kg/m^2^)
Underweight	<18.5
Normal	18.5–24.9
Overweight	25.0–29.9
Obese	≥30.0

**Table 3:** The demographic profile of soccer athletes.

Variable	Min	Max	Mean (±SD)
Age (yrs.)	18	28	21.6 (±2.01)
Period involved in soccer (yrs.)	1	4	3.2 (±1.09)
Duration spent during training (hrs.)	1	2	1.4 (± 0.49)

**Table 4:** Energy and macronutrients intake of athletes.

Variable	Min	Max	Mean (±SD)
Energy (kcal/kg/day)	5.4	297.3	39.6 ± 12.3
CHO (g/kg/day)	1.5	9.9	5.0 ± 1.5
Protein (g/kg/day)	0.5	2.9	1.4 ± 0.4
Fat (g/kg/day)	0.2	3.1	1.2 ± 0.6

**Table 5:** Relationship between BMI and dietary intake.

Variable	BMI Cat	Min	Max	Mean ± SD	*p*-Value
Energy intake (KJ/kg/day)	Underweight	44.7	68.5	58.1 ± 12.2	0.383
	Normal	13	69.1	38.8 ± 12.7	
	Overweight	120.6	183	37.7 ± 23.2	
CHO (g/kg/day)	Underweight	18	9	8.2 ± 1.0	0.261
	Normal	1.5	9.6	4.9 ± 1.6	
	Overweigh	1.6	5.7	4.2 ± 1.6	
Protein (g/kg/day)	Underweight	1.4	2.8	1.9 ± 0.7	0.543
	Normal	0.5	2.8	1.3 ± 0.5	
	Overweight	1.6	2.6	1.3 ± 0.2	
Fat (g/kg/day g)	Underweight	0.9	2.0	1.6 ± 0.5	0.179
	Normal	0.2	3.0	1.2 ± 0.440.8	
	Overweight	1.2	1.7	1.4 ± 0.17	

## Data Availability

The data presented in this study are available on request from the corresponding author.
